# Decreased Expression of Alpha-L-Fucosidase Gene *FUCA1* in Human Colorectal Tumors

**DOI:** 10.3390/ijms140816986

**Published:** 2013-08-19

**Authors:** Olalla Otero-Estévez, Mónica Martínez-Fernández, Lorena Vázquez-Iglesias, María Páez de la Cadena, Francisco J. Rodríguez-Berrocal, Vicenta S. Martínez-Zorzano

**Affiliations:** 1 Department of Biochemistry, Genetics and Immunology, University of Vigo, Campus As Lagoas Marcosende s/n, 36310 Vigo, Spain; E-Mails: olalla.otero@uvigo.es (O.O.-E.); lorena.vazquez@uvigo.es (L.V.-I.); mpaez@uvigo.es (M.P.C.); berrocal@uvigo.es (F.J.R.-B.); 2 Unit of Molecular Oncology, Department of Basic Research, Centro de Investigaciones Energéticas, Medioambientales y Tecnológicas (CIEMAT), 28040 Madrid, Spain; E-Mail: monica.martinez@ciemat.es

**Keywords:** alpha-l-fucosidase, colorectal cancer, *FUCA1*, RT-qPCR

## Abstract

In previous studies we described a decreased alpha-l-fucosidase activity in colorectal tumors, appearing as a prognostic factor of tumoral recurrence. The aim of this work was to extend the knowledge about tissue alpha-l-fucosidase in colorectal cancer by quantifying the expression of its encoding gene *FUCA1* in tumors and healthy mucosa. *FUCA1* mRNA levels were measured by RT-qPCR in paired tumor and normal mucosa tissues from 31 patients. For the accuracy of the RT-qPCR results, five candidate reference genes were validated in those samples. In addition, activity and expression of alpha-l-fucosidase in selected matched tumor and healthy mucosa samples were analyzed. According to geNorm and NormFinder algorithms, *RPLP0* and *HPRT1* were the best reference genes in colorectal tissues. These genes were used for normalization of *FUCA1* expression levels. A significant decrease of more than 60% in normalized *FUCA1* expression was detected in tumors compared to normal mucosa (*p =* 0.002). Moreover, a gradual decrease in *FUCA1* expression was observed with progression of disease from earlier to advanced stages. These findings were confirmed by Western blot analysis of alpha-l-fucosidase expression. Our results demonstrated diminished *FUCA1* mRNA levels in tumors, suggesting that expression of tissue alpha-l-fucosidase could be regulated at transcriptional level in colorectal cancer.

## 1. Introduction

Colorectal cancer (CRC) is the third most common form of cancer in the Western world. There are approximately one million new cases worldwide every year and overall survival is less than 60% in most countries [1], mainly due to the lack of reliable markers and symptoms previous to the development of metastasis.

Aberrant glycosylation is a hallmark of cancer, reflecting cancer-specific changes in glycan metabolism pathways such as altered expression of glycosyltransferases and glycosidases involved in the biosynthesis and catabolism of glycoconjugates [2,3]. The occurrence of these cancer-associated modifications on glycoconjugates between tumor and normal cells provides the opportunity for discovering new biomarkers for cancer diagnosis and prognosis [4,5]. In CRC, qualitative, quantitative and distributive differences in the expression of glycoconjugates between adenocarcinoma**-**derived tissue and normal mucosa are well known [6–8]. Among these changes, increased fucosylation has been described and related with malignant transformation, invasion and metastasis [7,9,10]. Fucosylation is influenced by several factors, including the levels and activities of the enzymes involved, the levels of GDP-fucose and other monosaccharide-nucleotides and the transport of these substrates across the membranes of the endoplasmic reticulum and Golgi apparatus. Nowadays, the enzymatic mechanisms leading to the alteration of fucosylation in CRC are not completely understood. In this regard, it is important to consider that the turnover of fucose residues in glycoconjugates is achieved through the involvement not only of fucosyltransferases, but also of the exoglycosidase fucosidase. Alpha-l-fucosidase (EC 3.2.1.51) is a lysosomal enzyme that removes terminal l-fucose residues present on the oligosaccharide chains of glycoconjugates. Mammalian alpha-l-fucosidases are multimeric forms of glycoprotein subunits exhibiting optimal activity at acidic pH values [11]. In humans, this enzyme is encoded by two genes: *FUCA1* (1p34), which codes the tissue enzyme, and *FUCA2* (6q24), which leads to plasma alpha-l-fucosidase [12].

The significance of tissue alpha-l-fucosidase in human metabolism is clearly shown in the genetic neurovisceral storage disease fucosidosis, caused by a defect of the *FUCA1* gene [13]. On the other hand, alterations in the activity levels of this enzyme have been described in different human malignancies. A diminished fucosidase activity has been reported in hepatocarcinoma [14,15] whereas an increased fucosidase activity has been found in endometrial, ovarian and cervical cancer [16,17] as well as in thyroid and gastric tumors [18]. Regarding CRC, a previous study from our laboratory demonstrated a decrease in the levels of alpha-l-fucosidase activity and amount of the enzymatic protein in tumoral tissue compared to healthy mucosa [19]. Moreover, this decrease seemed to be related to the progression of the disease, and tissue alpha-l-fucosidase activity appeared as a good independent prognostic factor of tumoral recurrence in CRC [20].

Based on those previous data, the aim of this study was to get insights into the knowledge of tissue alpha-l-fucosidase expression at gene level in CRC by quantifying *FUCA1* expression in tumors and paired healthy mucosa samples, and to determine whether the differences in the levels of alpha-l-fucosidase found in our previous works come from this molecular level. Among the currently used methods to measure gene expression, reverse transcription quantitative real-time PCR (RT-qPCR) represents a suitable technology that is being increasingly utilized in clinical assays, and was the method of choice in this study for the quantification of *FUCA1* expression. Previously, in order to ensure the accuracy of the RT-qPCR results, validation of the best reference genes in the same colorectal samples was carried out.

## 2. Results and Discussion

In this work, we have quantified by RT-qPCR the expression of *FUCA1*, the gene encoding tissue alpha-l-fucosidase, in tumor and matched normal mucosa samples from 31 colorectal cancer patients. RT-qPCR has become a commonly used method for assessing gene expression due to its high sensitivity, accuracy and reproducibility [21]. In order to obtain biologically meaningful results with this technique, data normalization against suitable internal reference gene(s) is required to correct non biological sample-to-sample variations. An ideal reference gene should be expressed at constant level in all the tested samples and pass through the same steps of analysis as the gene to be quantified [22]. Nowadays it is clear that there is no single and universal reference gene and that the inappropriate choice of reference genes can lead to misinterpretation of results [23]. Therefore, a previous analysis and validation of the best reference genes must be carried out for particular tissues or cell types and specific experimental designs. In our case, five genes commonly used as reference genes in the literature were chosen to be validated in our samples: *B2M* (beta-2-microglobulin), *GAPDH* (glyceraldehyde-3-phosphate dehydrogenase), *HPRT1* (hypoxanthine-guanine phosphoribosyltransferase), *PPIA* (peptidyl-prolyl *cis*-trans isomerase A) and *RPLP0* (60S acidic ribosomal protein P0). Genes involved in independent pathways were selected to minimize the effects of co-regulation.

### 2.1. Specificity of Primers and PCR Amplification Efficiency

First, the amplification specificity for each primer pair was verified by visualizing the amplicon of expected size on 2% agarose gels and confirmed by DNA sequencing (results not shown). Amplification efficiencies (*E*) for all primer pairs were determined by the standard curve method as described in the experimental section. The *E* values obtained were in the recommended range (90%–110%), and ranged from 90.61% for *HPRT1* to 106.24% for *FUCA1* (Table 1).

### 2.2. Expression Stability of Candidate Reference Genes in Colorectal Tissues

The raw quantification cycle (Cq) values for the five candidate reference genes were extracted from the data output file of StepOne thermal cycler and are inversely proportional to the initial mRNA amount present in the samples. The genes with higher expression in colorectal tissues were *RPLP0* and *PPIA* while the gene with the lowest expression was *HPRT1* (Table 1). These Cq data were transformed into relative quantities following the efficiency adjusted Delta Delta Cq method [24] and then analyzed by the geNorm [25] and NormFinder [26] algorithms in order to select the most stable reference genes in our samples. Among the five potential reference genes tested, those ranked in the top by both algorithms, and therefore considered as the most stable genes in colorectal tissues, were *RPLP0* and *HPRT1* (Table 2). This pair of genes was used for normalization of *FUCA1* expression levels in each sample.

*RPLP0* codifies a ribosomal protein that is a component of the 60S subunit. This gene has been chosen as reference gene in other studies using human tissues like small intestine [27], ovarian tissues [28] and ocular surface epithelium [29]. The gene *HPRT1*, that encodes the enzyme hypoxanthine-guanine phosphoribosyltransferase, has been validated as one of the reference genes in different human normal cells [30] and tumors such as glioblastoma [31] and hepatocarcinoma [32]. In addition, both genes have been chosen also as the best reference genes in colorectal tissues in previous studies [33,34].

### 2.3. Expression of the Target Gene *FUCA1* in Tumor and Normal Mucosa from CRC Patients

We quantified mRNA expression of the target gen *FUCA1* and the two selected reference genes, *RPLP0* and *HPRT1*, by RT-qPCR in paired tumor and normal mucosa samples from 31 CRC patients.

The expression (Cq value) of *FUCA1* in each sample was normalized to the geometric mean of reference genes applying the algorithm designed by Hellemans *et al.* [35]. Then, the normalized relative expression (NRQ) of *FUCA1* in matched tumor and healthy mucosa samples was compared by applying the non-parametric Wilcoxon matched-pairs signed rank test (Table 3).

As shown in Table 3, when all the colorectal cancer patients were considered as a group, a significant decrease of more than 60% (*p* = 0.002) in *FUCA1* expression was detected in tumoral tissue in comparison with normal mucosa. Then, subjects were subdivided according to the Dukes’ classification and *FUCA1* expression values in tumor and normal mucosa were compared. For each cancer subgroup *FUCA1* levels were always lower in tumoral tissue than in healthy mucosa, and a statistically significant reduction was observed for patients at stages A and B.

On the other hand, only considering tumoral samples, *FUCA1* expression was also compared among Dukes’ stages. In this case, a gradual decrease in the levels of *FUCA1* was observed with progression of the disease from earlier (stage A) to more advanced stages (stage C), although these differences were not statistically significant (Kruskal–Wallis test, *p* = 0.386), probably due to the small number of patients at each Dukes’ stage. This result is in agreement with our previous study describing that alpha-l-fucosidase activity was lower in tumors at advanced stages (Dukes’ stage C) than at early stages (Dukes’ stage A) [20]. We also compared the NRQ of *FUCA1* between Dukes’ A + B tumors (median: 0.00600; range: 0.00017–0.10387) and Dukes’ C tumors (median: 0.00315; range 0.00035–0.01510). In this case lower *FUCA1* expression values were found in the last group but the decrease was not statistically significant (Mann-Whitney *U*-test, *p* = 0.177) probably due to the great variability of *FUCA1* expression values among Dukes’ B tumors. In conclusion, according to our results and taking into account that no Dukes’ D stage patients were included in the studied group, a correlation between the decrease in *FUCA1* expression and tumor Dukes’ stage could not be established. There was no association between *FUCA1* expression levels and other features of tumors such as histological differentiation degree or tumor location (Table 4).

### 2.4. Validation of the RT-qPCR Data: Expression of Alpha-l-Fucosidase Protein

**Figure 1:**
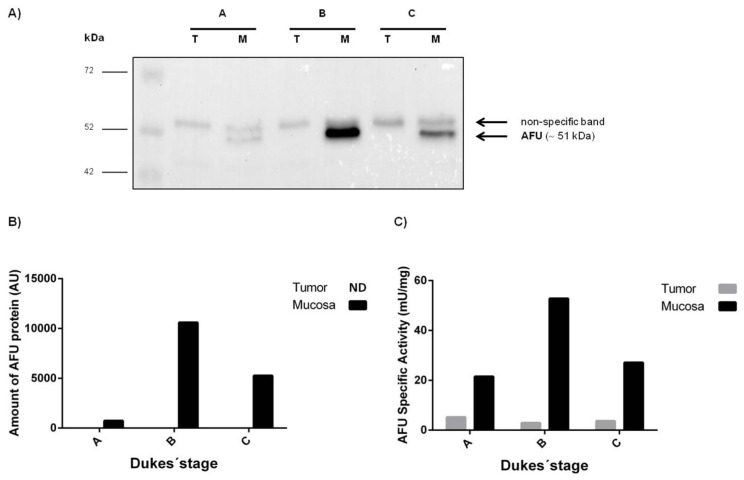
Expression and activity of AFU in colorectal cancer patients in different Dukes’ stages (stages A, B and C); (**A**) Immunodetection of AFU by Western blot in tumor (T) and healthy mucosa (M); each lane was loaded with 60 μg protein. The upper band is a non-specific band cross-reacting with the secondary antibody; (**B**) Quantification of AFU protein band in the same samples; this band was not detectable in tumor samples; (**C**) AFU specific activity in the same paired tumor and mucosa samples. (AFU: alpha-l-fucosidase, AU: Arbitrary Units, ND: Not Detectable).

In order to confirm the results found by RT-qPCR, we selected paired healthy mucosa and tumor samples from patients in different Dukes’ stages, evaluated the amount of alpha-l-fucosidase (AFU) protein by Western blot and determined the specific activity of this enzyme. Western blot analysis of AFU expression revealed one protein band of about 51 kDa corresponding to human AFU in the mucosa samples whereas the same band was not detected in the tumoral samples, under these conditions (Figure 1A,B). Consistent with the results obtained in the Western blot, the levels of AFU specific activity were lower in tumor than in normal mucosa, being the differences higher in the case of stage B and lower in the case of stage A (Figure 1C). Overall, these results demonstrated that AFU protein expression was reduced in the tumoral tissue in comparison with the normal mucosa, in agreement with our finding that show a decreased *FUCA1* expression in colorectal tumors.

Our results about *FUCA1* gene expression in colorectal tumors are in agreement with our previous study carried out with a different group of colorectal cancer patients [19]. In that work, we hypothesized that the reduction in alpha-l-fucosidase activity and amount of the enzymatic protein that we found in colorectal tumors could be a result of modulation of its synthesis and/or degradation. Our present data indicate that those changes in the concentration of alpha-l-fucosidase protein in colorectal tumors could be due to a decreased expression of its encoding gene *FUCA1*.

To date, although alterations in the activity of tissue alpha-l-fucosidase have been described in different human tumors [14–18], none of these studies have analyzed the expression of the *FUCA1* gene. Currently, we cannot determine which molecular mechanisms are leading to the decrease in *FUCA1* expression that we found in colorectal tumors. On one hand, it is well known the importance of transcription factors that bind to the promoter region of genes and modify their expression pattern. However, we did not find any studies about the transcription factors involved in the regulation of *FUCA1* expression. On the other hand, microRNAs which are short non-coding RNA molecules involved in regulating gene expression have been proposed as possible biomarkers in colorectal cancer. However, to the best of our knowledge, no microRNAs targeting *FUCA1* have been described until now. Another possible mechanism causing the down-regulation of *FUCA1* expression could be related to an aberrant methylation in tumors. Considering that the promoter region of this gene contains many GpC islands [36], susceptible to be methylated, a possible explanation could be a down-regulation of its expression caused by its aberrant methylation in tumors. Actually in cancer the epigenetic regulation through DNA methylation of genes involved in protein glycosylation is well known [37,38]. For instance, the epigenetic silencing of several fucosylation-related genes has been demonstrated in different cancer cell lines [39]. However, to the best of our knowledge, there are no previous studies analyzing the methylation of *FUCA1* gene in colorectal cancer. Because of this, futures analyses will be carried out to study the possible epigenetic regulation of *FUCA1* expression in colorectal tumors.

## 3. Experimental Section

### 3.1. Patients

For this study, 31 patients operated on for CRC at “Complejo Hospitalario Universitario de Vigo” (CHUVI, Vigo, Spain) were included. Written informed consent was obtained from patients or guardians, and anonymity was warranted through the use of clinical history numbers. None of the patients received therapy before surgery. The clinical pathological data of the patients are listed in Table 5. Primary adenocarcinoma samples as well as histologically normal mucosa, at least 10 cm from the tumor, were obtained by surgical resection and stored at −85 °C. The samples of normal mucosa and tumor tissue obtained from resection specimens were evaluated independently by two pathologists.

### 3.2. RNA Extraction

Total RNA was isolated from tissue samples using Tripure Isolation Reagent (Roche Diagnostics, Indianapolis, IN, USA) according to the manufacturer’s instructions, and treated with DNase I (Fermentas, Hanover, MD, USA) to remove any possible residual genomic DNA. The integrity of RNA samples was checked by agarose gel electrophoresis, visualizing two intact ribosomal 18S and 28S RNA bands. Concentration and purity of RNA samples were checked by measuring the absorbance A260/A280 ratio in a NanoDrop 2000 Spectrophotometer (Thermo Fisher Scientific, Wilmington, DE, USA). This ratio was between 1.90 and 2.0 for all the samples included in this study.

### 3.3. cDNA Synthesis

One μg of total RNA was reverse transcribed to cDNA with the Transcriptor First Strand cDNA Synthesis Kit (Roche Applied Science, Mannheim, Germany), using poli-dT primer. RNA samples were denatured for 10 min at 65 °C, following 30 min at 55 °C for reverse transcriptase reaction in a final volume of 20 μL (4 μL reaction buffer 5×, 2 μL dNTPs 10 μM, 0.5 μL reverse transcriptase 20 U/μL and 0.5 μL RNase inhibitor 40 U/μL). Finally the enzymes were inactivated at 85 °C for 5 min. Until use, cDNA samples were stored at −20 °C.

### 3.4. Primers for the Target Gene *FUCA1* and Candidate Reference Genes

The genes *B2M*, *GAPDH*, *HPRT1*, *PPIA* and *RPLP0* were tested as candidate reference genes in colorectal tissues. Primers for *RPLP0* were described previously [27]. Primers for the rest of reference genes as well as for the target gene *FUCA1* were designed using the program Primer3 (http://frodo.wi.mit.edu/primer3/, Whitehead Institute for Biomedical Research, Cambridge, MA, USA), taking into account primer-dimer formation, self-priming formation, and primer melting temperature. The sequences of all the primer pairs used are summarized in Table 6.

The specificity of each primer pair was verified by visualizing the amplicon size on 2% agarose gels and confirmed then by sequencing.

### 3.5. Quantitative Real-Time PCR (qPCR)

qPCR was performed on a StepOne thermal cycler (Applied Biosystems, Foster City, CA, USA) using SYBR green PCR master mix (Roche Dignostics, Indianapolis, IN, USA). First, the amplification efficiency of each primer pair was calculated as follows: qPCR was performed for each gene using serial dilutions of cDNA. Then the Cq values obtained were plotted against the logarithm of the amount of cDNA for the preparation of a standard curve. The slope of this standard curve was used for calculating PCR amplification efficiency (E) according to the equation *E* = 10^−1/slope^ [40].

The qPCR reaction was carried out using a 48-well plate MicroAmp optical (Applied Biosystems, Foster City, CA, USA). In each well, 1 μL of cDNA (1 μg of cDNA) and 9 μL of reaction mixture consisting of 5 μL of 2× FastStar Universal SYBR Green Master (Roche Diagnostics, Indianapolis, IN, USA), 0.5 μL of each primer (10 μM), and 3 μL of sterile water were added. The PCR reactions started with incubation for 2 min at 50 °C and 10 min at 95 °C, followed by 40 cycles consisting of 15 s at 95 °C and 60 s at 60 °C. The specificity of the amplified product was verified by melting curve analysis showing single peaks corresponding to unique amplicons. Non-template controls were included for quality control purposes. All cDNA samples were tested in triplicate from the same RNA preparation and the mean Cq values were used for further analysis.

### 3.6. Western Blot

For Western blot analysis, 60 μg of protein from colorectal tissues were separated by SDS-PAGE using a 10% resolving gel. All samples were diluted into sample buffer that contained 5% beta-mercaptoethanol and then heated at 95 °C for 5 min. After electrophoresis, proteins were transferred onto PVDF (Immobilon-P; Millipore, Madrid, Spain) for 1 h at 100 mA in a semidry blotting apparatus (Bio-Rad, Hercules, CA, USA). The membrane was blocked with 5% non-fat milk in Tris-buffered saline (TBS) containing 0.1% Tween 20 (TBST) for 2 h at room temperature. For immunoblotting, the membrane was incubated overnight at 4 °C with mouse monoclonal anti-FUCA1 IgG (ABCAM, Cambridge, UK) diluted 1/50 in blocking solution. After washing 6 times with TBST to remove unbound antibody, the membrane was then incubated for 45 min with rabbit anti-Mouse IgG conjugated with horseradish peroxidase (HRP) (Sigma Chemical, St. Louis, MO, USA) diluted 1/50,000 in the blocking solution described above. The membrane was washed and then drained to remove excess liquid. Detection of the protein of interest was obtained by chemiluminescence detection using the ECL kit (GE Healthcare, New Haven, CT, USA). To control the background emission and the possible non-specific bands, a parallel membrane was incubated with the secondary antibody only (data not shown). The ECL signal was visualized using a digital camera-based imaging system (ChemiDoc™, Biorad, Hercules, CA, USA). Signal intensities of bands were quantified by Image Lab software (Bio-Rad). Loading control was performed by staining the membrane with Coomassie Brilliant Blue (R-250) (data not shown).

### 3.7. Alpha-l-Fucosidase Activity and Protein Determination in Tumor and Normal Colorectal Mucosa

Colorectal tissues were sonicated (Sonifier Cell Disruptor 450, Branson, Danbury, CT, USA) at 4 °C in 10 mM, pH 6.5 phosphate buffer (8 mL/mg tissue) containing a cocktail of protease inhibitors (Complete TM Mini tablets, Roche, Indianapolis, IN, USA). Alpha-l-fucosidase activity was measured as previously described [19], using the synthetic substrate 4-methyl-umbelliferyl-α-l-fucopyranoside (4-MU-fucoside) (Sigma Chemical, St. Louis, MO, USA) at a final concentration of 0.36 mM, and expressed as specific activity (mU/mg). Protein concentration was assessed employing the Bradford micro assay (Bio-Rad, Hercules, CA, USA).

### 3.8. Statistical Analyses

All statistical analyses were performed with the SPSS software for MS Windows (v.15.0, SPSS Inc., Chicago, IL, USA). Normality of variables was assessed according to Kolmogorov-Smirnov test. For the comparison of non-normal distributed values, the non-parametric two-tailed Wilcoxon matched-pairs signed-ranks test, the Kruskal–Wallis test and Mann-Whitney *U*-test were used. *p* values ≤ 0.05 were considered statistically significant.

## 4. Conclusions

In conclusion, our present data demonstrate a reduction in the mRNA of *FUCA1* in colorectal tumors, suggesting that the expression of tissue alpha-l-fucosidase could be regulated at the transcriptional level. These results provide valuable information on the mechanisms involved in the alteration of alpha-l-fucosidase expression in colorectal cancer and explain our previous findings describing a reduced alpha-l-fucosidase activity and enzymatic protein content in the tumoral tissue. Moreover, a future study exploring the utility of *FUCA1* gene as a prognostic marker for colorectal cancer is warranted.

## Figures and Tables

**Table 1:** Amplification efficiencies and quantification cycle (Cq) values of candidate reference genes and *FUCA1* in colorectal tissues.

Gen	Amplification efficiency (%)	*R*^2^	Slope	Cq median	Cq range
*B2M*	101.00	0.989	−3.2992	25.54	19.23–33.80
*GAPDH*	93.40	0.997	−3.4913	25.68	18.28–33.75
*HPRT1*	90.61	0.993	−3.5696	31.25	23.09–36.28
*PPIA*	104.37	0.999	−3.2214	23.32	17.82–31.01
*RPLP0*	92.95	0.994	−3.5034	23.65	18.33–29.64
*FUCA1*	106.24	0.997	−3.1810	27.96	23.08–36.12

**Table 2:** Stability ranking of candidate reference genes in colorectal tissues.

Stability ranking	GeNorm	NormFinder
Best pair	*RPLP0/HPRT1*	*RPLP0/HPRT1*
Ranking	Gen	Gen
1	*RPLP0*	*HPRT1*
2	*HPRT1*	*RPLP0*
3	*PPIA*	*GAPDH*
4	*B2M*	*PPIA*
5	*GAPDH*	*B2M*

**Table 3:** *FUCA1* expression in tumor and normal mucosa from colorectal patients.

Dukes’ stage	*N*	Tumor		Normal mucosa		*p*^a^
	
		Median	Range	Median	Range	
Stages A + B + C	31	0.00452	0.00017–0.10387	0.01184	0.00009–0.20345	<0.01
Stage A	8	0.00650	0.00017–0.00867	0.02324	0.00009–0.09498	<0.05
Stage B	11	0.00452	0.00048–0.10387	0.04132	0.00101–0.20345	<0.05
Stage C	12	0.00315	0.00035–0.01510	0.00688	0.00016–0.15438	NS *

a Wilcoxon matched-pairs signed rank test;

* NS: No statistically significant.

**Table 4:** Association of *FUCA1* expression levels with features of tumors.

Features of tumors		*N*	Median	Range	*p*^a^
Tumor differentiation	Well	4	0.00373	0.00017–0.00750	0.192
	Moderate	21	0.00416	0.00035–0.09684	
	Poor	6	0.00788	0.00386–0.10387	

Tumor location	Right colon	12	0.00508	0.00173–0.01510	0.388
	Left colon	14	0.00575	0.00017–0.10387	
	Rectum	5	0.00060	0.00035–0.09684	

a Kruskal-Wallis test.

**Table 5:** Clinical-pathological data of the colorectal cancer (CRC) patients.

Clinical-pathological variable		Number of patients
Gender	Male	21
	Female	10

Age	Range	56–89
	Mean ± SD	70 ± 9.41

Tumor Dukes’ stage	A	8
	B	11
	C	12

Tumor differentiation	Well	4
	Moderate	21
	Poor	6

Tumor location	Right colon	12
	Left colon	14
	Rectum	5

**Table 6:** Primer sequences for the candidate reference genes and the target gene *FUCA1*.

Gen	Protein name	GenBank ID	Amplicon size (bp)	Primers sequences (5′to 3′)
*B2M*	beta-2-microglobulin	NM_004048	228	F:TTTCATCCATCCGACATTGA R:CCTCCATGATGCTGCTTACA
*GAPDH*	glyceraldehyde-3-phosphate dehydrogenase	NM_002046	238	F:GAGTCAACGGATTTGGTCGT R:TTGATTTTGGAGGGATCTCG
*HPRT1*	hypoxanthine-guanine phosphoribosyltransferase	NM_000194	248	F:CCCCACGAAGTGTTGGATA R:AGCAGATGGCCACAGAACT
*PPIA*	peptidyl-prolyl cis-trans isomerase A	NM_021130	188	F:CAAGAAGATCACCATTGCT R:AGGGAACAAGGAAAACAT
*RPLP0*	60S acidic ribosomal protein P0	NM_001002	140	F:GCAATGTTGCCAGTGTCTG R:GCCTTGACCTTTTCAGCAA
*FUCA1*	tissue alpha-L-fucosidase	NM_000147	190	F:AGTCACCCTGTTGCCTATGG R:TTTGGCGCTTTTAGATTGCT
